# Cost-effectiveness of HLX01 (Hanlikang^®^) vs. rituximab combined with CHOP in treatment-naive diffuse large B-Cell lymphoma: a partitioned survival model analysis

**DOI:** 10.3389/fphar.2025.1498735

**Published:** 2025-10-01

**Authors:** Chang Wang, Yuanqing Huang, Langling Rao, Chunling Yu, Yingxin Zhang, Yingtao Lin

**Affiliations:** ^1^ Department of Lymphoma and Head and Neck Tumors, Clinical Oncology School of Fujian Medical University, Fujian Cancer Hospital, Fuzhou, Fujian, China; ^2^ Hospital Vice-Superintendent, Fujian Maternity and Child Health Hospital, College of Clinical Medicine for Obstetrics and Gynecology and Pediatrics, Fujian Medical University, Fuzhou, Fujian, China; ^3^ Department of Gynecological-Surgical Oncology, Clinical Oncology School of Fujian Medical University, Fujian Cancer Hospital, Fuzhou, Fujian, China; ^4^ Clinical Medical Research Center, Clinical Oncology School of Fujian Medical University, Fujian Cancer Hospital, Fuzhou, Fujian, China

**Keywords:** diffuse large B-cell lymphoma, cost-effectiveness, partitioned survival model, Hanlikang, rituximab

## Abstract

**Background:**

This study evaluates the cost-effectiveness of Hanlikang (HLX01), a biosimilar of rituximab, compared to rituximab (MabThera), both combined with the cyclophosphamide, doxorubicin, vincristine, and prednisone (CHOP) regimen, for treatment-naive diffuse large B-cell lymphoma (DLBCL) patients. With biosimilars becoming more prominent in oncology, understanding their economic impact is crucial for optimizing treatment strategies and healthcare resource allocation.

**Methods:**

A partitioned survival model was built using data from the HLX01-NHL03 trial, analyzing clinical outcomes in three health states—progression-free survival, progressive disease, and terminal state—over a 10-year time horizon. The incremental cost-effectiveness ratio (ICER) and quality-adjusted life years (QALYs) were compared across transplant-eligible and non-transplant-eligible patient subgroups. Sensitivity analyses were performed to confirm the robustness of the model.

**Results:**

Over 10 years, Hanlikang-CHOP (H-CHOP) patients gained 7.11 QALYs, compared to 6.50 QALYs for Rituximab-CHOP (R-CHOP). The ICER for transplant-eligible patients ranged from US$ 36,386.92 (CNY 263,215.70) to US$ 38,379.79 (CNY 277,631.72) per QALY, depending on the treatment. In non-transplant-eligible patients, the ICER was between US$ 7,079.15 (CNY 51,209.16) and US$ 17,094.61 (CNY 123,658.99) per QALY. Chimeric antigen receptor T-cell (CAR-T) therapy significantly increased the ICER to US$ 356,793.77 (CNY 2,580,974.77). Sensitivity analyses confirmed survival duration and drug costs as key factors.

**Conclusion:**

Hanlikang offers a cost-effective alternative to rituximab in specific patient populations, particularly those not eligible for transplant. However, its economic benefits diminish in more complex treatment scenarios, such as those involving CAR-T therapy or novel agents.

## 1 Introduction

Diffuse large B-cell lymphoma (DLBCL) is the most common subtype of non-Hodgkin’s lymphoma (NHL). It accounts for 25%–30% of cases worldwide and over one-third of lymphoid neoplasms in China (GBD, 2016 Causes of Death Collaborators, 2017). Although DLBCL is an aggressive malignancy, it responds well to the R-CHOP regimen (rituximab, cyclophosphamide, doxorubicin, vincristine, and prednisone), which remains the standard of care for newly diagnosed patients (Coiffier and Sarkozy, 2016). Rituximab is a chimeric monoclonal antibody targeting the cluster of differentiation (CD20) antigen expressed on the surface of B lymphocytes. It exerts its therapeutic effects by inducing the destruction of malignant B lymphocytes through mechanisms such as complement-dependent cytotoxicity, antibody-dependent cell-mediated cytotoxicity, phagocytosis, and apoptosis (Cerny et al., 2002). The pivotal LNH-98.5 trial demonstrated that R-CHOP significantly improves complete response rates and overall survival (OS) compared to CHOP alone, establishing it as the standard of care for newly diagnosed DLBCL patients (Coiffier et al., 2010). Recent large phase III studies have attempted to enhance R-CHOP efficacy by adding targeted agents, such as bortezomib (Offner et al., 2015), lenalidomide (Nowakows et al., 2016), or ibrutinib (Younes et al., 2019). However, none of these approaches have significantly improved outcomes. The POLARIX trial (NCT03274492) demonstrated that the combination of rituximab, cyclophosphamide, doxorubicin, and prednisone (R-CHP) with the antibody-drug conjugate polatuzumab-vedotin (Pola) significantly enhanced progression-free survival (PFS) in previously untreated patients with diffuse large B-cell lymphoma (DLBCL) who had an International Prognostic Index (IPI) score of 2-5, compared to the standard R-CHOP regimen (Tilly et al., 2022). However, the 2-year OS remains immature. Consequently, R-CHOP continues to be the standard first-line treatment for DLBCL.

In China, the clinical benefits of R-CHOP have been well-documented, with first-line treatment achieving high response rates in DLBCL patients. However, the high cost of rituximab, a chimeric anti-CD20 monoclonal antibody, has limited its accessibility, imposing a financial burden on many patients and restricting its widespread use (Khor et al., 2014).

To address this issue, biosimilars of rituximab have been developed, providing comparable efficacy and safety at a reduced cost. These biosimilars are anticipated to enhance the treatment of DLBCL from a health economics standpoint. Hanlikang (HLX01), developed by Shanghai Henlius Biotech, was approved in February 2019 and entered the market. This product is the first rituximab biosimilar approved in China (Shi et al., 2020). Phase 1 and phase 3 trials have shown that Hanlikang has similar pharmacokinetics and pharmacodynamics to the reference rituximab, and produces comparable clinical outcomes. In the HLX01-NHL03 phase 3 study, Hanlikang combined with CHOP (H-CHOP) showed comparable efficacy to R-CHOP in treatment-naive DLBCL patients with low to intermediate risk, with similar overall response rates (Qin et al., 2024).

Given the comparable clinical outcomes and the potential for cost savings, Hanlikang represents a promising alternative to rituximab in the treatment of DLBCL. Therefore, this study aimed to evaluate the cost-effectiveness of Hanlikang compared to rituximab, both in combination with the CHOP regimen, for treatment-naive DLBCL patients in China, using a partitioned survival model. This analysis is intended to provide evidence-based insights that could support treatment optimization and improve patient access in the context of China’s healthcare system.

## 2 Materials and methods

### 2.1 Target population

The target population for this study was based on the criteria used in the HLX01-NHL03 trial, a Phase III, multicenter, double-blind, randomized controlled study conducted across 27 hospitals in China, including Fujian Cancer Hospital. The study included patients with newly diagnosed, treatment-naive DLBCL, confirmed by histology and CD20 positivity. Eligible participants were aged 18–80 years, with an IPI score of 0–2, an Eastern Cooperative Oncology Group (ECOG) performance status of 0–2, and a life expectancy of at least 6 months. Additional requirements included adequate hematologic, liver, and renal function, as well as negative human immunodeficiency virus (HIV), hepatitis C virus (HCV), and hepatitis B virus (HBV) status, or low viral loads if positive. Exclusion criteria encompassed prior NHL treatment, central nervous system (CNS) involvement, double- or triple-hit diffuse large B-cell lymphoma, significant comorbidities, or active infections. All participants provided informed consent prior to enrollment.

### 2.2 Intervention

The participants were divided into two groups. The experimental group received Hanlikang combined with the CHOP regimen, while the control group received Rituximab (MabThera) combined with the CHOP regimen. Treatment was administered in 3-week (21-day) cycles, for a total of 6 cycles. On Day 1 (D1) of each cycle, Hanlikang or MabThera was administered intravenously at a dose of 375 mg/m^2^. On Day 2 (D2), the CHOP regimen was administered, consisting of cyclophosphamide 750 mg/m^2^, doxorubicin 50 mg/m^2^, and vincristine 1.4 mg/m^2^ (with a maximum dose of 2 mg). From Day 2 to Day 6 (D2-D6), prednisone 100 mg was administered daily.

### 2.3 Model construction

**FIGURE 1:**
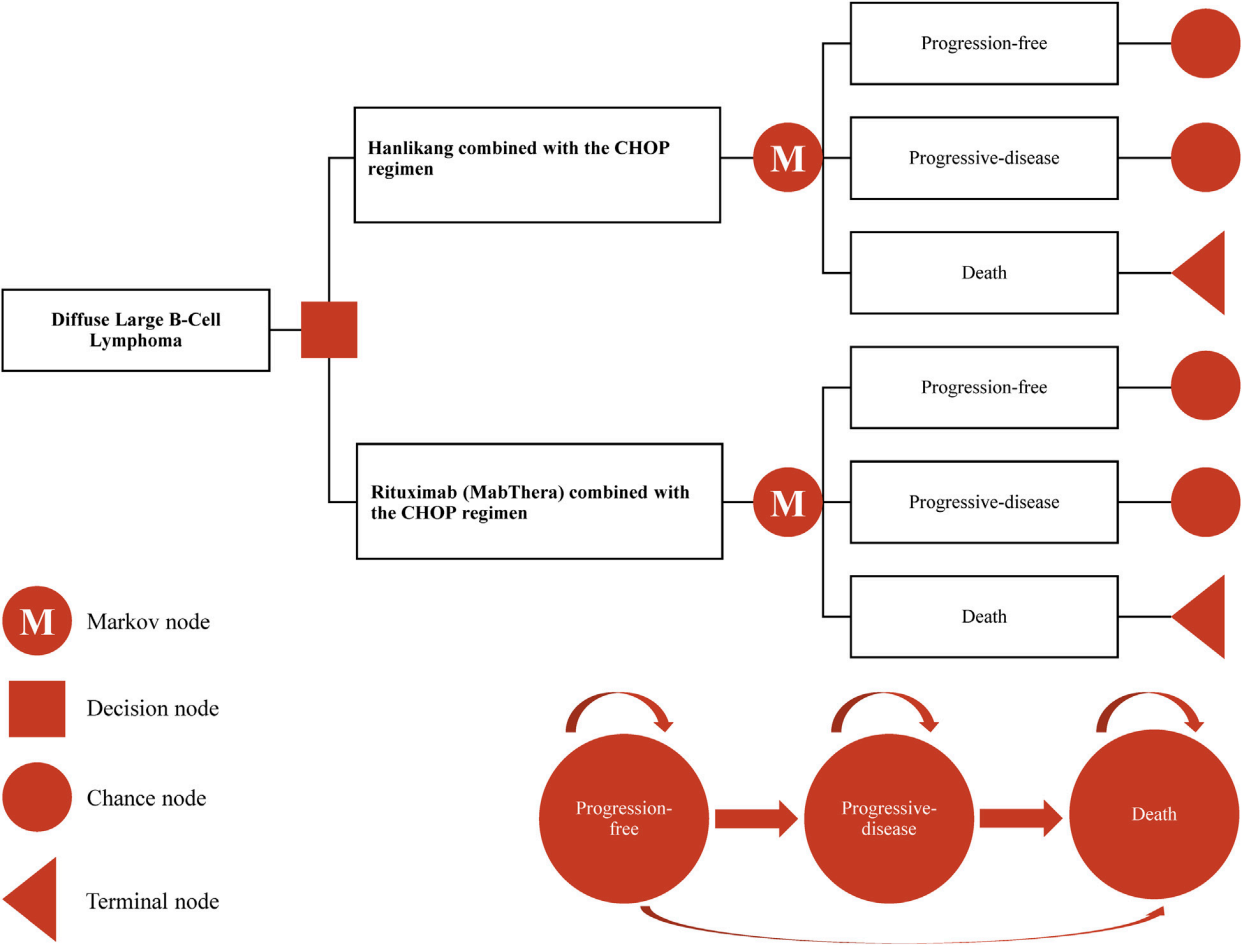
Profile of the partitioned survival model for the HLX01-NHL03 trial.

The cost-effectiveness analysis was conducted using a partitioned survival model (Shim et al., 2024), based on data from the HLX01-NHL03 trial. This model, commonly used in metastatic oncology research, evaluates both financial and clinical outcomes. It incorporates three distinct health states (Yang et al., 2022): the progression-free state (from patient entry until disease progression), the progressive disease (PD) state (the period during which the patient remains alive after disease progression), and the terminal state (as illustrated in Figure 1). In our model, each cycle was set to 21 days, with a time horizon of 10 years. A half-cycle correction was applied to account for the timing of transitions between health states. Key outputs from the model include costs, quality-adjusted life years (QALYs), and the incremental cost-effectiveness ratio (ICER).

### 2.4 Cost assessment

The cost assessment included a comprehensive evaluation of clinical expenses associated with cancer treatment, covering drug acquisition, laboratory tests, radiological evaluations, medication administration, post-progression treatment, treatment-related adverse events (AEs), and end-of-life care. These costs were classified as direct medical expenditures and converted to U.S. dollars using the April 2024 exchange rate: 1 USD = 7.2338 CNY. Financial data were sourced from credible institutions, including the National Health Commission of China, the Health Commission of Fujian Province, and expert consensus. Detailed cost parameters are provided in Table 1.

**TABLE 1:** Key model inputs.

Input parameters	Base case value	Lower bound	Upper bound	Distribution	Source
Log-normal OS survival model					
H-CHOP	meanlog (λ) = 3.284; sdlog (γ) = 1.938	-	-	Log-Normal	Qin et al. (2024)
R-CHOP	meanlog (λ) = 2.925; sdlog (γ) = 1.843	-	-	Log-Normal	Qin et al. (2024)
Log-Normal PFS survival model					
H-CHOP	meanlog (λ) = 2.998; sdlog (γ) = 1.884	-	-	Log-Normal	Qin et al. (2024)
R-CHOP	meanlog (λ) = 3.007; sdlog (γ) = 2.195	-	-	Log-Normal	Qin et al. (2024)
Drug acquisition, US$					
Hanlikang (Shanghai Henlius Biotech, Inc.) per 100 mg	193.09	154.47	231.71	Gamma	National Health Commission of China
MabThera (Roche Pharmaceuticals) per 100 mg	253.57	202.85	304.28	Gamma	National Health Commission of China
Cyclophosphamide (Baxter Oncology GmbH, Germany) per 1,000 mg	26.61	21.29	31.94	Gamma	National Health Commission of China
Epirubicin (Shandong New Era Pharmaceutical Co., Ltd.) per 10 mg	10.88	8.70	13.05	Gamma	National Health Commission of China
Vincristine (Hefei Yifan Biopharmaceutical Co., Ltd.) per 1 mg	38.29	30.63	45.95	Gamma	National Health Commission of China
Prednisone tablets (Fuzhou Haixin Pharmaceutical Co., Ltd.) per 5 mg	1.61	1.29	1.94	Gamma	National Health Commission of China
Acetaminophen (Shenyang Aojina Pharmaceutical Co., Ltd.) per 100 mg	5.52	4.42	6.62	Gamma	National Health Commission of China
Diphenhydramine (Hebei Meitu Pharmaceutical Co., Ltd.) per 20 mg	4.70	3.76	5.64	Gamma	National Health Commission of China
Dexamethasone (Anhui Changjiang Pharmaceutical Co., Ltd.) per 5 mg	0.99	0.80	1.19	Gamma	National Health Commission of China
Drug administration, per cycle, US$					
Drug administration Hospitalization	19.35	15.48	23.22	Gamma	Local medical data
Drug administration Preventive medication	67.58	54.06	81.09	Gamma	Local medical data
Drug administration Infusion	5.26	4.21	6.31	Gamma	Local medical data
Laboratory and imaging examination, US$					
12-lead ECG	3.73	2.99	4.48	Gamma	Fujian Provincial Health Commission
Hematology	3.46	2.76	4.15	Gamma	Fujian Provincial Health Commission
Serum chemistry	23.50	18.80	28.20	Gamma	Fujian Provincial Health Commission
Hepatic and Renal Function Tests + Electrolytes	18.07	14.45	21.68	Gamma	Fujian Provincial Health Commission
Immunoglobulin levels	8.72	6.97	10.46	Gamma	Fujian Provincial Health Commission
Coagulation Function Tests	9.20	7.36	11.04	Gamma	Fujian Provincial Health Commission
Urinalysis	4.15	3.32	4.98	Gamma	Fujian Provincial Health Commission
Echocardiography	39.12	31.30	46.95	Gamma	Fujian Provincial Health Commission
Contrast-enhanced CT	308.81	247.05	370.58	Gamma	Fujian Provincial Health Commission
Costs of AE (AEs with an incidence ≥10%), US$					
Leukopenia	118.70	94.96	142.44	Gamma	expert consensus
Neutropenia	3,881.64	3,105.31	4,657.97	Gamma	expert consensus
Anemia	63.60	50.88	76.32	Gamma	expert consensus
Thrombocytopenia	334.78	267.82	401.74	Gamma	expert consensus
Nausea	273.00	218.40	327.60	Gamma	expert consensus
Elevated ALT	1,372.98	1,098.39	1,647.58	Gamma	expert consensus
Decreased appetite	273.00	218.40	327.60	Gamma	expert consensus
Alopecia	150.00	120.00	180.00	Gamma	expert consensus
Cough	65.52	52.42	78.62	Gamma	expert consensus
Vomiting	344.93	275.94	413.92	Gamma	expert consensus
Upper respiratory tract infection	1,991.14	1,592.91	2,389.37	Gamma	expert consensus
Hypokalemia	493.90	395.12	592.68	Gamma	expert consensus
Constipation	71.67	57.33	86.00	Gamma	expert consensus
Non-infectious pneumonia	1,338.40	1,070.72	1,606.08	Gamma	expert consensus
Diarrhea	290.65	232.52	348.78	Gamma	expert consensus
Febrile neutropenia	3,881.64	3,105.31	4,657.97	Gamma	expert consensus
Bone marrow failure (Pancytopenia)	6,641.76	5,313.41	7,970.11	Gamma	expert consensus
H-CHOP AE risks (AEs with an incidence ≥10%)					
Leukopenia	0.855	0.684	1.03	Beta	Qin et al. (2024)
Neutropenia	0.790	0.632	0.95	Beta	Qin et al. (2024)
Anemia	0.385	0.308	0.46	Beta	Qin et al. (2024)
Thrombocytopenia	0.170	0.136	0.20	Beta	Qin et al. (2024)
Nausea	0.230	0.184	0.28	Beta	Qin et al. (2024)
Elevated ALT	0.245	0.196	0.29	Beta	Qin et al. (2024)
Decreased appetite	0.160	0.128	0.19	Beta	Qin et al. (2024)
Alopecia	0.175	0.140	0.21	Beta	Qin et al. (2024)
Cough	0.155	0.124	0.19	Beta	Qin et al. (2024)
Vomiting	0.110	0.088	0.13	Beta	Qin et al. (2024)
Upper respiratory tract infection	0.095	0.076	0.11	Beta	Qin et al. (2024)
Hypokalemia	0.140	0.112	0.17	Beta	Qin et al. (2024)
Constipation	0.135	0.108	0.16	Beta	Qin et al. (2024)
Non-infectious pneumonia	0.095	0.076	0.11	Beta	Qin et al. (2024)
Diarrhea	0.080	0.064	0.10	Beta	Qin et al. (2024)
Febrile neutropenia	0.025	0.020	0.03	Beta	Qin et al. (2024)
Bone marrow failure (Pancytopenia)	0.025	0.020	0.03	Beta	Qin et al. (2024)
R-CHOP AE risks (AEs with an incidence ≥10%)					
Leukopenia	0.885	0.708	1.06	Beta	Qin et al. (2024)
Neutropenia	0.840	0.672	1.01	Beta	Qin et al. (2024)
Anemia	0.360	0.288	0.43	Beta	Qin et al. (2024)
Thrombocytopenia	0.095	0.076	0.11	Beta	Qin et al. (2024)
Nausea	0.245	0.196	0.29	Beta	Qin et al. (2024)
Elevated ALT	0.190	0.152	0.23	Beta	Qin et al. (2024)
Decreased appetite	0.210	0.168	0.25	Beta	Qin et al. (2024)
Alopecia	0.170	0.136	0.20	Beta	Qin et al. (2024)
Cough	0.130	0.104	0.16	Beta	Qin et al. (2024)
Vomiting	0.150	0.120	0.18	Beta	Qin et al. (2024)
Upper respiratory tract infection	0.145	0.116	0.17	Beta	Qin et al. (2024)
Hypokalemia	0.085	0.068	0.10	Beta	Qin et al. (2024)
Constipation	0.125	0.100	0.15	Beta	Qin et al. (2024)
Non-infectious pneumonia	0.120	0.096	0.14	Beta	Qin et al. (2024)
Diarrhea	0.110	0.088	0.13	Beta	Qin et al. (2024)
Febrile neutropenia	0.030	0.024	0.04	Beta	Qin et al. (2024)
Bone marrow failure (Pancytopenia)	0.025	0.020	0.03	Beta	Qin et al. (2024)
Terminal cost, US$					
End-of-life care	1,036.80	829.44	1,244.16	Gamma	Local data
Utility value					
Progression-free disease	0.83	0.66	1.00	Beta	Kambhampati et al. (2022)
Progressive disease	0.63	0.50	0.76	Beta	Kambhampati et al. (2022)
Disutility due to AEs with an incidence ≥10%	−0.42	−0.34	−0.50	Beta	Li et al. (2022)
Discount rate	0.05	0	0.08	Beta	Liu et al. (2020)

Abbreviations: OS, overall survival; PFS, Progression-Free Survival; H-CHOP, hanlikang, Cyclophosphamide, Doxorubicin, Vincristine, and Prednisone; R-CHOP, MabThera, Cyclophosphamide, Doxorubicin, Vincristine, and Prednisone; ECG, electrocardiogram; CT, computed tomography; AE, adverse events; ALT, alanine aminotransferase.

The main cost components evaluated were as follows:

Drug Acquisition Costs: 1) Hanlikang: US$ 139.09 per 100 mg (Shanghai Henlius Biotech, Inc.). 2) MabThera: US$ 253.57 per 100 mg (Roche Pharmaceuticals). 3) Cyclophosphamide: US$ 26.61 per 1,000 mg (Baxter Oncology GmbH). 4) Epirubicin: US$ 10.88 per 10 mg (Shandong New Era Pharmaceutical Co., Ltd.). 5) Vincristine: US$ 38.29 per 1 mg (Hefei Yifan Biopharmaceutical Co., Ltd.). 6) Prednisone tablets: US$ 1.61 per 5 mg (Fuzhou Haixin Pharmaceutical Co., Ltd.).

Dosage and Patient Characteristics: 1) Drug dosages and strengths were based on the HLX01-NHL03 trial. 2) An average body surface area of 1.73 m^2^ was assumed, estimated from the China Statistical Yearbook 2022.

Drug Administration and Hospitalization Costs: 1) Included hospitalization, nursing care, and infusion logistics. 2) Prophylactic medications were administered 60 min before drug infusion, including oral acetaminophen 30 min prior, followed by intravenous diphenhydramine hydrochloride and dexamethasone. 3) Drug wastage was accounted for by rounding drug quantities to the nearest vial size, with surplus drugs discarded post-infusion according to standard practice.

Laboratory and Imaging Costs: 1) Electrocardiograms (ECGs), hematology tests, serum chemistry assessments, and urinalysis. 2) Contrast-enhanced computed tomography (CT) scans (neck, chest, abdomen, and pelvis) every two treatment cycles, until disease progression or patient death.

Adverse Event (AE) Management Costs: 1) Costs were calculated based on the 2023 edition of the charging standards issued by the Fujian Provincial Health Commission. 2) Included AEs with incidence rates >10% and other common AEs. 3) Assuming that the AEs occurred during the initial treatment cycle. 4) Cost estimates derived from expert consensus. The detailed management strategies for AEs are provided in Supplementary Table S1.

Post-Progression Treatment Costs: Included consultations, salvage regimens after progression or relapse.

End-of-Life Care Costs: Estimated based on the same 2023 edition of the Fujian Provincial Health Commission’s charging standards, which were still in use during the study period.

### 2.5 Utility scores

Utility scores quantify the quality of life associated with different health states. Although the HLX01-NHL03 trial did not provide specific patient utility scores, we used quality of life data from existing literature as benchmarks for utility scores in the cost-effectiveness evaluation of DLBCL treatments. In our model, these scores were based on established utility evaluations for DLBCL. Specifically, the utility score for PFS was set at 0.83 (Kambhampati et al., 2022), for PD at 0.63 (Kambhampati et al., 2022), and for death at 0 (Kambhampati et al., 2022). Additionally, we accounted for the negative impact of AEs on a patient’s quality of life, incorporating these effects as reductions in utility scores (Li et al., 2022). The key utility parameters are detailed in Table 1.

### 2.6 Sensitivity analyses

We conducted a deterministic sensitivity analysis on our model by systematically varying all input parameters within a range of ±20% (Mizuoka et al., 2023). By adjusting each parameter in isolation while keeping all other variables at their base values, we assessed the impact of individual parameters on the stability of the model. The annual discount rate for both costs and health outcomes was set at 5%, with a sensitivity range from 0% to 8% (Liu et al., 2020).

In addition, we performed a probabilistic sensitivity analysis using Monte Carlo simulations (Zhang et al., 2023; Lin et al., 2023). In this analysis, cost parameters were assumed to follow a gamma distribution, while utility parameters were modeled using a beta distribution (Briggs et al., 2012). For each simulation iteration, values for all parameters were simultaneously and randomly drawn from their respective distributions. After completing 10,000 iterations, we analyzed the combined impact of these parameter variations to evaluate the model’s robustness.

### 2.7 Scenario analysis

In our scenario analysis, we examined several potential treatment pathways for patients with DLBCL who experienced initial progression or relapse. The goal was to assess how these different treatment strategies might influence the model’s outcomes. For patients eligible for transplant, we considered two scenarios: 1) autologous stem cell transplantation (ASCT) after response to traditional salvage chemotherapy such as dexamethasone, high-dose cytarabine, and cisplatin (DHAP) regimen (van Imhoff et al., 2017) and 2) a combination of a novel agent with traditional salvage chemotherapy followed by ASCT, such as lenalidomide or acalabrutinib added to the DHAP regimen (Duel et al., 2021). For patients not eligible for transplant, we evaluated two alternative strategies: 1) traditional salvage chemotherapy using the DHAP regimen alone (Gisselbrecht et al., 2010) and 2) novel agent therapies, which included either the R2 regimen (lenalidomide plus rituximab) combined with a Bruton’s tyrosine kinase (BTK) inhibitor (acalabrutinib) or the polatuzumab vedotin, bendamustine, and rituximab (Pola-BR) regimen (Sehn et al., 2020). Additionally, for patients with primary refractory or early relapse (≤12 months post-first-line), regardless of transplant eligibility, we included the use of chimeric antigen receptor T-cell (CAR-T) therapy, specifically commercial products such as axicabtagene ciloleucel (Yescarta^®^, Fosun Kite) and relmacabtagene autoleucel (Carteyva^®^, JW Therapeutics) (Neelapu et al., 2017). This comprehensive analysis allowed us to understand the potential impact of various treatment strategies on the cost-effectiveness and overall outcomes of the model.

### 2.8 Statistical analysis

We used Get Data Graph Digitizer software (version 2.26) to extract survival curves from the HLX01-NHL03 trial data and reconstructed individual patient data using R software (version 4.2.2) to model patient survival rates across various distributions, including Weibull, log-normal, log-logistic, Gompertz, Gamma, and exponential. The most appropriate distribution was selected based on the lowest values of the Akaike Information Criterion (AIC) and Bayesian Information Criterion (BIC), supported by visual inspection and relevant literature. Ultimately, the log-normal distribution was chosen to model both PFS and OS curves for the patient groups (as illustrated in Supplementary Table S2). Calculations of costs and health outcomes for the three distinct health states, along with results from scenario and sensitivity analyses, were conducted using Excel (version 2019).

## 3 Results

### 3.1 Base-case analysis

Over a 10-year period, patients in the H-CHOP group consistently gained 7.11 QALYs, compared to 6.50 QALYs in the R-CHOP group, across various treatment scenarios. The incremental costs and ICERs varied depending on the treatment approach:

For patients eligible for transplant: Traditional salvage chemotherapy followed by a transplant using the DHAP regimen resulted in an incremental cost of US$ 21,969.00 and an ICER of US$ 36,386.92 (CNY 263,215.70)/QALY. When a novel agent, such as lenalidomide or acalabrutinib, was added to the regimen, the incremental cost increased to US$ 23,172.22, with an ICER of US$ 38,379.79 (CNY 27,7631.72)/QALY (as detailed in Table 2).

**TABLE 2:** Cost-effectiveness analysis results.

Treatment regimen	Total costs (US$)	Quality-adjusted life years (QALYs)	Incremental cost-effectiveness ratio (ICER, US$/QALY)
**Scenario 1:** Patients Eligible for Transplant - Traditional Salvage Chemotherapy with DHAP Regimen			
H-CHOP	94,467.21	7.11	36,386.92
R-CHOP	72,498.21	6.50	-
**Scenario 2:** Patients Eligible for Transplant - Novel Agent + DHAP Regimen			
H-CHOP	98,753.03	7.11	38,379.79
R-CHOP	75,580.80	6.50	
**Scenario 3:** Patients Not Eligible for Transplant - Traditional Salvage Chemotherapy with DHAP Regimen			
H-CHOP	31,438.75	7.11	7,079.15
R-CHOP	27,164.63	6.50	
**Scenario 4:** Patients Not Eligible for Transplant - Novel Agent Therapies (R2 + BTK Inhibitor or Pola-BR Regimen)			
H-CHOP	52,977.72	7.11	17,094.61
R-CHOP	42,656.66	6.50	
**Scenario 5**: Primary refractory or early relapse Patients - CAR-T Cell Therapy			
H-CHOP	783,525.17	7.11	356,793.77
R-CHOP	568,107.00	6.50	

Abbreviations: QALY, Quality-Adjusted Life Year; ICER, Incremental Cost-Effectiveness Ratio; H-CHOP, hanlikang, Cyclophosphamide, Doxorubicin, Vincristine, Prednisone; R-CHOP, MabThera, Cyclophosphamide, Doxorubicin, Vincristine, Prednisone; DHAP, dexamethasone, High-Dose Cytarabine, and Cisplatin; BTK, Bruton’s Tyrosine Kinase; Pola-BR, polatuzumab vedotin, Bendamustine, and Rituximab; CAR-T, Chimeric Antigen Receptor T-Cell.

For patients not eligible for transplant: Traditional salvage chemotherapy alone resulted in an incremental cost of US$ 4,274.11 and an ICER of US$ 7,079.15 (CNY 51,209.16)/QALY. When novel agent therapies were used, including the lenalidomide plus rituximab (R2) regimen combined with a BTK inhibitor or the Pola-BR regimen, the incremental cost was US$ 10,321.06, with an ICER of US$ 17,094.61 (CNY 123,658.99)/QALY (as detailed in Table 2).

For patients with primary refractory or early relapse: When treated with CAR-T cell therapy, the incremental cost was significantly higher at US$ 215,418.17, resulting in an ICER of US$ 356,793.77(CNY 2,580,974.77)/QALY (as detailed in Table 2).

### 3.2 Sensitivity analyses

#### 3.2.1 Deterministic sensitivity analyses

Across various treatment scenarios, one-way deterministic sensitivity analyses consistently showed that the model was most sensitive to the survival duration of the H-CHOP group. Additional key factors influencing the model outcomes included the survival duration of the R-CHOP group, the utility score for PFS, the costs of treatment after PD, and the acquisition costs of MabThera and Hanlikang.

**FIGURE 2:**
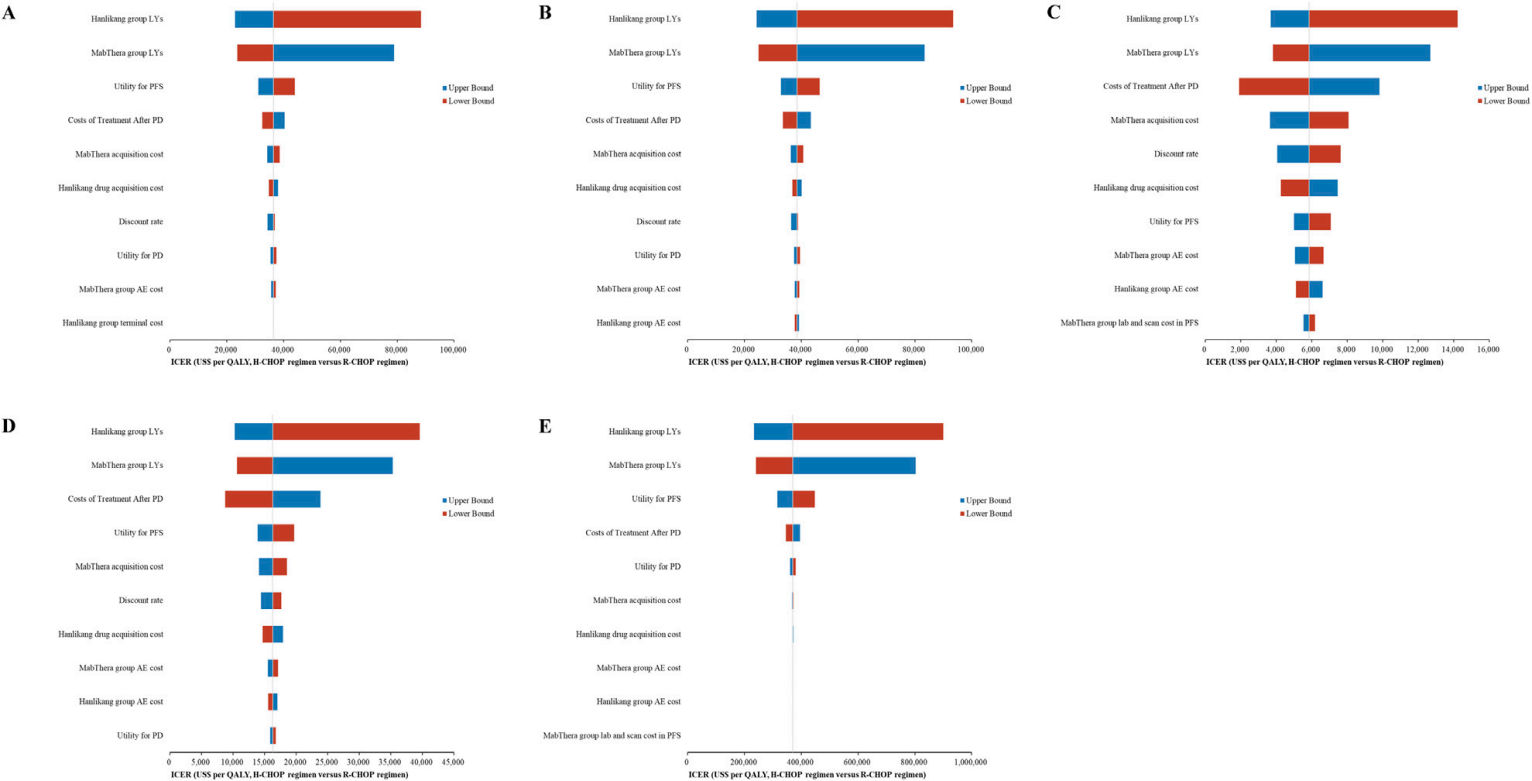
Tornado diagrams **(A-E)** depicting the Top 10 most influential parameters across five treatment scenarios: **(A)** Transplant-eligible with DHAP regimen; **(B)** Transplant-eligible with novel agent + DHAP; **(C)** Non-transplant-eligible with DHAP regimen; **(D)** Non-transplant-eligible with novel agent therapies; **(E)** CAR-T cell therapy scenario.

For patients eligible for transplant, treated with traditional salvage chemotherapy followed by a transplant using the DHAP regimen, ICER values ranged between US$ 23,000 (CNY 166,377.4) and US$ 89,000 (CNY 643,808.2) (as depicted in Figure 2A). When a novel agent was added to the salvage chemotherapy regimen, ICER values varied between US$ 24,000 (CNY 173,611.2) and US$ 93,000 (CNY 672,743.4) (as depicted in Figure 2B).

For patients not eligible for transplant, treated with traditional salvage chemotherapy alone, ICER values ranged from US$ 3,600 (CNY 26,041.68) to US$ 14,000 (CNY 101,273.2) (as depicted in Figure 2C). For those receiving novel agent therapies, ICER values fluctuated between US$ 10,000 (CNY 72,338) and US$ 40,000 (CNY 289,352) (as depicted in Figure 2D).

In the scenario involving CAR-T cell therapy for primary refractory or early relapse patients, ICER values varied widely, from US$ 230,000 (CNY 1,663,774) to US$ 900,000 (CNY 6,510,420) (as depicted in Figure 2E).

#### 3.2.2 Probabilistic sensitivity analyses

**FIGURE 3:**
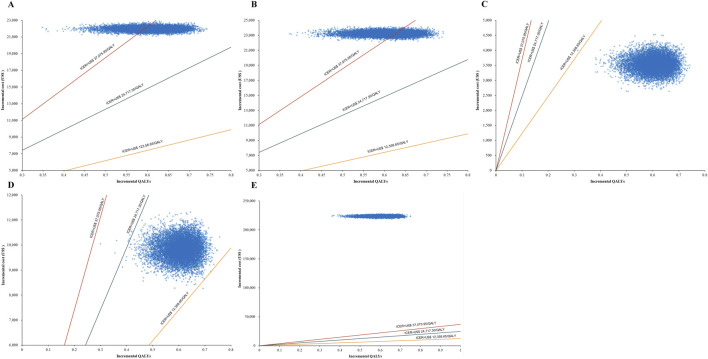
Scatter plots **(A-E)** representing Monte Carlo sensitivity analysis for Hanlikang versus rituximab across five treatment scenarios: **(A)** Transplant-eligible with DHAP regimen; **(B)** Transplant-eligible with novel agent + DHAP; **(C)** Non-transplant-eligible with DHAP regimen; **(D)** Non-transplant-eligible with novel agent therapies; **(E)** CAR-T cell therapy scenario.

**FIGURE 4:**
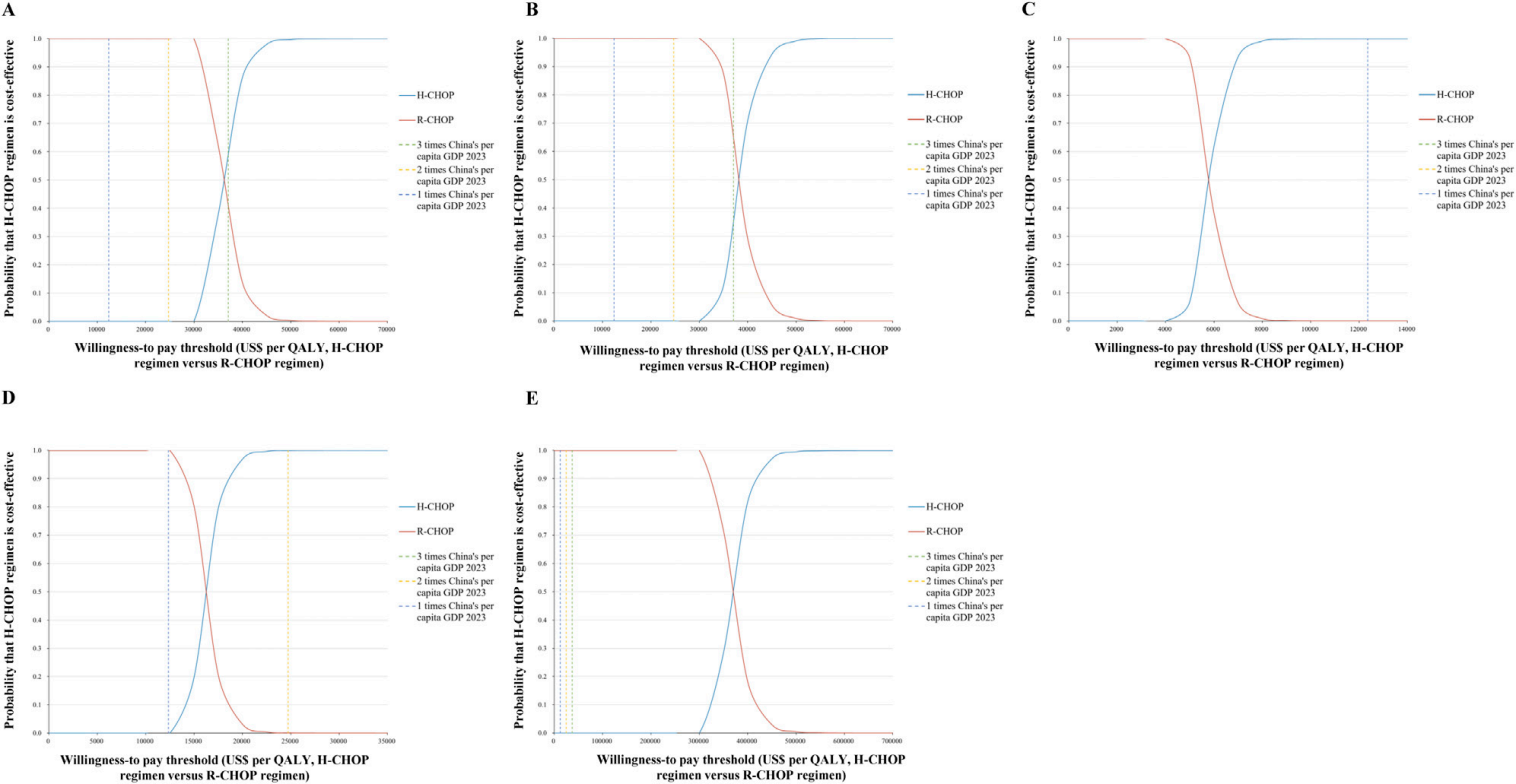
Cost-effectiveness acceptability curves **(A-E)** comparing Hanlikang plus CHOP versus rituximab plus CHOP across five treatment scenarios: **(A)** Transplant-eligible with DHAP regimen; **(B)** Transplant-eligible with novel agent + DHAP; **(C)** Non-transplant-eligible with DHAP regimen; **(D)** Non-transplant-eligible with novel agent therapies; **(E)** CAR-T cell therapy scenario.

The World Health Organization (WHO) recommends a willingness-to-pay (WTP) threshold set at three times the gross domestic product (GDP) *per capita* (Murray et al., 2000). As of 2023, China’s GDP *per capita* was US$ 12,358.65, setting the WTP threshold at US$ 37,075.95 (CNY 268,200.01)/QALY. To capture a broader range of economic conditions and reflect evolving health technology assessment (HTA) practices, we additionally conducted probabilistic sensitivity analyses using WTP thresholds of 1× and 2× China’s GDP *per capita* (US$12,358.65 (CNY 89,400.00) and US$24,717.30 (CNY 178,800.00) per QALY, respectively). The cost-effectiveness probabilities of H-CHOP compared with R-CHOP under these three WTP thresholds are summarized below:

For patients eligible for transplant receiving traditional salvage chemotherapy followed by a transplant using the DHAP regimen (dexamethasone, high-dose cytarabine, and cisplatin), the probability of H-CHOP being cost-effective was 0% at both the 1× and 2× GDP thresholds, and 63.44% at the 3× threshold (as depicted in Figures 3A, 4A).

For patients eligible for transplant receiving a combination of a novel agent with traditional salvage chemotherapy followed by transplant (e.g., lenalidomide or acalabrutinib added to the DHAP regimen), the cost-effectiveness probability of H-CHOP was 0% at both the 1× and 2× thresholds, and 38.33% at the 3× threshold (as depicted in Figures 3B, 4B).

For patients not eligible for transplant receiving traditional salvage chemotherapy using the DHAP regimen alone, the probability of H-CHOP being cost-effective was 99.99%, 100%, and 100% at the 1×, 2×, and 3× GDP thresholds, respectively (as depicted in Figures 3C, 4C).

For patients not eligible for transplant receiving novel agent therapies, including either the R2 regimen (lenalidomide plus rituximab) combined with a BTK inhibitor (acalabrutinib) or the Pola-BR regimen, the cost-effectiveness probabilities of H-CHOP were 0.02%, 99.91%, and 100% at the 1×, 2×, and 3× thresholds, respectively (as depicted in Figures 3D, 4D).

For patients with primary refractory or early relapse, regardless of transplant eligibility, who were treated with CAR-T cell therapy, H-CHOP was not found to be cost-effective under any WTP threshold, with a probability of 0% across all levels (as depicted in Figures 3E, 4E).

## 4 Discussion

With the expiration of patents on many originator biologics, biosimilars like HLX01 have emerged as cost-effective alternatives. Although not identical to reference products, biosimilars are approved based on rigorous demonstration of similarity in quality, safety, and efficacy (Nagle et al., 2003). HLX01 has shown bioequivalence to rituximab in both pharmacokinetics and clinical efficacy, as confirmed in Phase 1 and Phase 3 trials involving patients with CD20-positive B-cell lymphoma and DLBCL (Shi et al., 2020; Declerck et al., 2017; Shi et al., 2021). The key advantage of biosimilars lies in their potential to significantly reduce treatment costs while maintaining therapeutic outcomes, thereby improving accessibility in real-world healthcare settings, especially in countries like China where budget constraints remain a major concern.

These considerations are particularly relevant for low-income and resource-limited regions in western China, where affordability continues to be a major barrier to optimal care. By delivering similar clinical efficacy at a significantly lower cost, Hanlikang has the potential to enhance equity in cancer care delivery and expand treatment access for socioeconomically disadvantaged populations. Our previous real-world research has shown that following the inclusion of biologics such as rituximab and trastuzumab in China’s National Reimbursement Drug List (NRDL), and their subsequent integration into national volume-based procurement (VBP) initiatives, utilization rates increased substantially (Shang et al., 2023). Unlike traditional reimbursement-focused strategies, VBP reduces drug prices through bulk purchasing agreements, making essential medications more affordable—even for patients without insurance coverage. Consequently, Hanlikang’s competitive pricing, when coupled with policy mechanisms such as VBP, has the potential to significantly lower the financial barriers to DLBCL treatment across all socioeconomic groups (Xu et al., 2025).

Unlike many other malignancies, the incidence of diffuse large B-cell lymphoma (DLBCL) in China does not appear to vary significantly across different geographic or socioeconomic regions (Zheng et al., 2023). This epidemiological consistency suggests that the burden of disease is relatively uniform nationwide, regardless of local income levels. Consequently, interventions that enhance treatment accessibility—such as biosimilars like Hanlikang—have the potential to generate widespread public health benefits across all provinces. Our study demonstrates that for treatment-naive patients with DLBCL, the cost-effectiveness of the H-CHOP regimen compared to the R-CHOP regimen is highly dependent on patient survival duration and the subsequent treatment strategies required. At a willingness-to-pay (WTP) threshold of US$ 37,075.95 per QALY, equivalent to three times China’s *per capita* GDP, our findings indicate that H-CHOP is cost-effective in certain Monte Carlo iterations but not in others. This suggests that its cost-effectiveness may vary depending on the clinical context and healthcare environment. Specifically, while H-CHOP may be cost-effective in some scenarios, its cost-effectiveness may diminish as treatment complexity increases. Notably, even under more conservative economic assumptions—such as using a WTP threshold of one or two times *per capita* GDP—H-CHOP remained cost-effective in several key clinical scenarios.

For patients with relapsed and refractory DLBCL, ASCT is regarded as a well-established salvage consolidation therapy. This approach significantly prolongs PFS and OS by restoring normal hematopoietic and immune functions (Stiff et al., 2011). Currently, the utilization of ASCT in China is experiencing a year-on-year increase, with 98% of patients acquiring hematopoietic stem cells for transplantation through peripheral blood stem cells (PBSCs) (Yuan and Wang, 2017). Nonetheless, the proportion of lymphoma patients in China undergoing ASCT remains comparatively lower than that observed in European and American countries (Zhu, 2020). Several factors may contribute to this disparity, including economic underdevelopment, uneven advancement of regional healthcare facilities, the risk associated with the mobilization of hematopoietic stem cells from bone marrow to peripheral blood, and cultural differences in perceptions of life and death, which are influenced by Confucian philosophy. For those who either opt out of transplantation or are ineligible, H-CHOP demonstrated a clear cost-effectiveness advantage, whether combined with traditional salvage chemotherapy, such as the DHAP regimen, or with novel treatment strategies including the R2 regimen (lenalidomide plus rituximab) combined with a BTK inhibitor or the Pola-BR regimen. This supports the use of H-CHOP as a viable alternative to R-CHOP in these patient populations. In contrast, for transplant-eligible patients, cost-effectiveness outcomes were less favorable. When using traditional salvage chemotherapy followed by ASCT (i.e., the DHAP regimen), the probability of H-CHOP being more cost-effective than R-CHOP was 63.44% at the 3× GDP threshold, but 0% at both 1× and 2× thresholds. When novel agents such as lenalidomide or BTK inhibitors were added to the salvage regimen prior to transplant, this probability further declined to 38.33% at the 3× threshold. Furthermore, for patients with primary refractory or early relapse treated with CAR-T cell therapy, the significantly higher costs result in H-CHOP not being cost-effective compared to R-CHOP.

These findings align with the study by Swetha Kambhampati et al., which assessed the cost-effectiveness of polatuzumab vedotin combined with chemoimmunotherapy in untreated DLBCL using a Markov model (Kambhampati et al., 2022). Their research also concluded that the cost-effectiveness of polaR-CHP compared to R-CHOP is highly sensitive to patient survival duration and varies depending on clinical settings and treatment strategies across different healthcare environments. Given the heterogeneous nature of DLBCL patients and the emphasis on personalized treatment, with numerous new and advanced therapies being integrated into clinical practice, it is essential to differentiate between various clinical contexts and healthcare environments to accurately evaluate the cost-effectiveness of H-CHOP relative to R-CHOP. This differentiation can help guide payers, clinicians, and patients in selecting the most appropriate DLBCL treatment strategies (Ren et al.).

We observed that the pivotal Phase III clinical trial of HLX01 exclusively enrolled low-risk DLBCL patients with IPI scores ranging from 0 to 2 (Shi et al., 2020). However, the approved indications for HLX01 in China encompass DLBCL patients with IPI scores spanning from 0 to 5. Despite some authors arguing that the IPI has diminished in predictive utility in the rituximab era, it continues to serve as a significant instrument for the risk stratification of DLBCL patients (Fu et al., 2008; Sehn et al., 2007). Notably, higher IPI scores correlate with a poorer prognosis and a more intricate treatment regimen (International Non-Hodgkin’s Lymphoma Prognostic Factors Project, 1993). A study conducted by Zhao Weili analyzed clinical data from a substantial cohort of 2,592 newly diagnosed diffuse large B-cell lymphoma (DLBCL) patients, among whom 1,233 underwent DNA sequencing to identify oncogenic mutations (Wang et al., 2024). Based on the IPI score, patients were stratified into four groups: the low-risk group (IPI 0–1) comprising 1,229 cases (47.4%), the low to medium risk group (IPI 2) comprising 521 cases (20.1%), the high to medium risk group (IPI 3) comprising 493 cases (19.0%), and the high-risk group (IPI 4–5) comprising 349 cases (13.5%). The 5-year overall survival rates for these groups were 89.7%, 71.7%, 55.3%, and 41.6%, respectively. The mutation frequencies of PIM1, MYD88, CD79B, CREBBP, TBL1XR1, FAS, MYC, and CD79A were significantly elevated in patients with an IPI of 4-5, whereas the mutation frequencies of CARD11, PRDM1, and FBXW7 were notably increased in patients with an IPI of 3–5. Additionally, the MYD88L265P mutation, either alone or in conjunction with the CD79B mutation, is more prevalent in patients with an IPI of 4–5. ZHAO’s study revealed differences in prognosis and different mutation frequencies among DLBCL populations with different IPI scores. Consequently, while our findings are most directly applicable to patients with IPI scores 0–2 based on the HLX01-NHL03 trial, it is also important to consider their implications for patients with higher IPI scores (Cerny et al., 2002; Coiffier et al., 2010; Offner et al., 2015). For this higher-risk subgroup, National Comprehensive Cancer Network (NCCN) guidelines (National Comprehensive Cancer Network (NCCN), 2025) still recommend R-CHOP as preferred regimen for first-line therapy. Other recommended regimens include dose-adjusted EPOCH (etoposide, prednisone, vincristine, cyclophosphamide, doxorubicin) combined rituximab and Pola-R-CHP in some cases (IPI>1, Stage I-II). Therefore, the R-CHOP regimen, for which H-CHOP offers a biosimilar alternative, remains highly relevant in this patient population. These DLBCL patients with high IPI scores may necessitate augmented treatment strategies, including novel targeted agents, beyond conventional chemotherapy, due to the presence of oncogenic mutations (e.g., double- or triple-hit lymphoma, or specific oncogenic mutations noted by Zhao Weili, which were not the focus of the HLX01-NHL03 trial population) in tumor-associated genes (Reiter et al., 2019). In such scenarios, these patients’ first-line treatment with R-CHOP/H-CHOP may produce different results and they may be suitable for regimens such as pola-R-CHP. They may also need to receive expensive subsequent treatments (such as CAR-T therapy). Based on the aforementioned rationale and the results of the cost-effectiveness analysis conducted in this study, it is recommended that caution be exercised when administering HLX01 to patients with DLBCL who have an IPI score greater than 2. A high IPI score may signify an elevated risk of recurrence and increased treatment complexity, rendering the H-CHOP regimen less cost-effective compared to R-CHOP. Further studies or real-world evidence in these high-risk DLBCL populations will be essential to refine the economic evaluation of H-CHOP in a wider range of clinical practice. Additionally, given that HLX01 has been available on the market for just over 5 years, its long-term safety and efficacy remain uncertain and warrant further observation.

While H-CHOP shows potential as a cost-effective alternative to R-CHOP in several scenarios, R-CHOP continues to hold significant cost-effectiveness advantages, particularly in the context of CAR-T therapy. Two randomized phase III studies have shown that second-line CAR-T therapy improves progression-free survival (PFS) compared to standard therapy for patients with primary refractory or early relapsed DLBCL (Locke et al., 2022; Kamdar et al., 2022), leading to U.S. Food and Drug Administration (FDA) approval of second-line axicabtagene ciloleucel (axi-cel) for these patients (Locke et al., 2022). As a result, we anticipate that the cost-effectiveness of first-line R-CHOP may remain challenging for H-CHOP to surpass, especially as second-line CAR-T therapy becomes more widely adopted (Kambhampati et al., 2022). However, in settings where CAR-T therapy is less expensive or not widely available, R-CHOP may lose its cost-effectiveness advantage. Additionally, if CAR-T therapy costs can be reduced through alternative payment mechanisms (e.g., reimbursement only for successful infusions) (Wu et al., 2023) or increased competition with the introduction of more products (e.g., allogeneic off-the-shelf CAR-T therapies) (Kelkar et al., 2023), the potential for H-CHOP to emerge as a cost-effective alternative to R-CHOP would significantly increase.

This study recognizes several limitations that require consideration. First, our model is mainly based on clinical trial data, which may introduce subtle biases and uncertainties. It is also important to note that the long-term therapeutic outcomes of combining Hanlikang with Rituximab in treating DLBCL are still being investigated. A longer follow-up period could help refine and update the data, leading to clearer insights. Second, our model does not fully account for certain aspects, such as costs associated with specific adverse events. While excluding these factors might slightly affect the results, sensitivity analyses suggest they are unlikely to significantly influence the primary conclusions within the model’s variability range. Third, utility values play a key role in pharmacoeconomic studies, and due to the lack of trial-specific quality-of-life data, our analysis relied on published utility values for DLBCL. Even though the sensitivity analysis suggests that utility values for progression-free and progressive disease could affect the outcomes, adjustments within acceptable limits would not drastically alter the cost-effectiveness results. Fourth, our analysis was conducted from the perspective of the Chinese healthcare system, which includes only direct medical costs incurred by public providers. As such, indirect societal costs—such as productivity loss, time costs, and caregiver burden—were not included. This is a notable limitation, particularly given that DLBCL often affects individuals of working age, and such costs can be substantial. However, due to the lack of robust local data on these factors, they were not incorporated into the present model. Future research should consider evaluating H-CHOP from a broader societal perspective to provide a more comprehensive assessment of its economic impact. Fifth, this study did not include formal stakeholder engagement during model development. While frontline clinicians from the HLX01-NHL03 trial were consulted to ensure alignment with real-world practice, no structured input was obtained from patients or healthcare payers. This limits the breadth of perspectives represented in the model. Future research would benefit from incorporating broader stakeholder input to enhance relevance and applicability. Lastly, A key consideration is that the HLX01-NHL03 trial only enrolled treatment-naive DLBCL patients with low IPI (IPI 0–2). Therefore, understanding how our cost-effectiveness findings for H-CHOP apply to high-risk DLBCL patients (IPI 3–5) needs careful thought. These higher-risk patients often have poorer prognoses and may require more complex subsequent treatments. The economic outcomes for H-CHOP in this specific subgroup were not directly modeled from trial data covering the entire IPI 3-5 range and therefore require further investigation.

## 5 Conclusion

This study demonstrates that Hanlikang, while offering comparable clinical efficacy to rituximab, has the potential to be a cost-effective alternative in specific DLBCL patient populations and clinical settings. The economic advantage of Hanlikang is most apparent in patients not eligible for transplant, particularly those receiving traditional salvage chemotherapy or novel agent regimens. However, in transplant-eligible patients, the cost-effectiveness of Hanlikang diminishes, especially when novel agents are incorporated into the treatment regimen. Additionally, the high cost of CAR-T therapy in DLBCL patients with primary refractory or early relapse complicates the cost-effectiveness of Hanlikang compared to rituximab. These findings support the continued integration of biosimilars like Hanlikang into national reimbursement and procurement frameworks in China, with the potential to improve treatment affordability, reduce regional disparities in access, and enhance the overall sustainability of cancer care delivery.

## Data Availability

The original contributions presented in the study are included in the article/Supplementary Material, further inquiries can be directed to the corresponding author.
