# Supplementary material for: Cost-effectiveness of HLX01 (Hanlikang®) vs. rituximab combined with CHOP in treatment-naive diffuse large B-Cell lymphoma: a partitioned survival model analysis
Source: Front Pharmacol. 2025 Oct 1;16:1498735. doi: 10.3389/fphar.2025.1498735 (PMC12521807; doi:10.3389/fphar.2025.1498735)
Supplement: Supplementary file 1 [file Table1.docx]

| **Table S1 Management Strategies for Treatment-Emergent Adverse Events (AEs)** | | | | |
| --- | --- | --- | --- | --- |
| **Adverse Events (AEs)** | **Grade 1 AE Management** | **Grade 2 AE Management** | **Grade 3 AE Management** | **Grade 4 AE Management** |
| Leukopenia | No treatment required | Oral leukocyte-promoting agents | G-CSF | Emergency hospitalization + G-CSF + prophylactic antibiotic therapy |
| Neutropenia | No treatment required | Oral leukocyte-promoting agents | G-CSF | Emergency hospitalization + G-CSF + prophylactic antibiotic therapy |
| Anemia | No treatment required | Erythropoietin administration | Blood transfusion | Emergency hospitalization + blood transfusion + anti-infective therapy + supportive care |
| Thrombocytopenia | No treatment required | Oral thrombopoietic agents | Thrombopoietin (TPO) and interleukin therapy | Emergency hospitalization + platelet transfusion therapy + thrombopoietin (TPO) |
| Nausea | No treatment required | Prokinetic agents and progestins | Nasogastric feeding, total parenteral nutrition, or hospitalization | N/A |
| Elevated ALT | Hepatoprotective agents | Multiple types of hepatoprotective agents and etiology-specific treatment | Hepatoprotective agents, etiology-specific treatment, enteral nutrition, and correction of hypoproteinemia | Hepatoprotective agents, etiology-specific treatment, enteral nutrition, and correction of hypoproteinemia |
| Decreased appetite | No treatment required | Oral nutritional supplements and progestins | Nasogastric feeding or total parenteral nutrition | Emergency hospitalization, total parenteral nutrition, and supportive care |
| Alopecia | No treatment required | Use of wigs or hairpieces | N/A | N/A |
| Cough | Over-the-counter medication | Prescription medication | Prescription medication | N/A |
| Vomiting | Oral monotherapy with antiemetics | Intravenous antiemetics and oral nutritional supplementation | Combination antiemetic therapy and electrolyte supplementation | Emergency hospitalization, combination antiemetic therapy, and supportive care |
| Upper respiratory tract infection | N/A | Oral antibiotics, antifungal or antiviral therapy | Intravenous administration of antibiotics, antifungals, or antiviral agents | Emergency hospitalization, combination drug therapy, and supportive care |
| Hypokalemia | No treatment required | Oral potassium supplementation | Hospitalization | Emergency admission, intravenous potassium supplementation, and supportive care |
| Constipation | Occasional use of stool softeners, laxatives, dietary adjustments, or enemas | Continued use of laxatives or enemas | Manual disimpaction | Emergency admission |
| Non-infectious pneumonia | No intervention needed if asymptomatic | Intervention required | Intervention and oxygen support required | Emergency admission and urgent treatment |
| Diarrhea | Adsorptive antidiarrheal agents such as montmorillonite | Montmorillonite and gut microbiota-regulating agents | Rehydration, maintenance of water-electrolyte balance, montmorillonite, and gut microbiota-regulating agents | Emergency admission, urgent treatment, massive fluid replacement, appropriate diuresis, and supportive care |
| Febrile neutropenia | N/A | N/A | Hospitalization + G-CSF + anti-infective therapy | Emergency hospitalization + G-CSF + anti-infective therapy + resuscitation support |
| Bone marrow failure (Pancytopenia) | No treatment required | Mainly symptomatic treatment | Supportive care, blood transfusion, injection of erythropoietin and thrombopoietin, G-CSF, infection and bleeding prevention | Emergency hospitalization + supportive care, blood transfusion, injection of erythropoietin and thrombopoietin, G-CSF, infection and bleeding prevention |
| Abbreviations: G-GSF, Granu1ocyte colony-stimu1ating factor; ALT, Alanine aminotransferase; N/A: Not Applicable, indicates that the specific grade is not defined for this adverse event in CTCAE(Common Terminology Criteria for Adverse Events) v4.03. †The grading of treatment-emergent adverse events described in this section is based on the CTCAE version 4.03. | | | | |
