# Supplementary material for: Cost-effectiveness of HLX01 (Hanlikang®) vs. rituximab combined with CHOP in treatment-naive diffuse large B-Cell lymphoma: a partitioned survival model analysis
Source: Front Pharmacol. 2025 Oct 1;16:1498735. doi: 10.3389/fphar.2025.1498735 (PMC12521807; doi:10.3389/fphar.2025.1498735)
Supplement: Supplementary file 2 [file Table2.docx]

| **Table S2 Summary of Statistical Goodness-of-fit of Partitioned Survival Model** | | | | | | |
| --- | --- | --- | --- | --- | --- | --- |
|  | **Exponential** | **Weibull** | **Gamma** | **Gompertz** | **log-Normal** | **log-Logistic** |
| **PFS Curve of H-CHOP** | | |  |  |  |  |
| AIC | 278.7731 | 280.5111 | 280.6295 | 278.5282 | **276.7835** | 279.6278 |
| BIC | 281.8293 | 286.6236 | 286.742 | 284.6407 | **282.896** | 285.7403 |
| **PFS curve of R-CHOP** | | |  |  |  |  |
| AIC | 319.3611 | 319.4706 | 319.6085 | 318.949 | **318.9208** | 319.1297 |
| BIC | 322.43 | 325.6084 | 325.7463 | 325.0869 | **325.0586** | 325.2675 |
| **OS Curve of H-CHOP** | | |  |  |  |  |
| AIC | 250.9719 | 252.9021 | 252.9335 | 252.421 | **250.8849** | 252.5679 |
| BIC | 254.0281 | 259.0146 | 259.046 | 258.5335 | **256.9974** | 258.6804 |
| **OS curve of R-CHOP** | | |  |  |  |  |
| AIC | 299.4209 | 301.4066 | 301.4177 | 301.1428 | **299.9606** | 301.0488 |
| BIC | 302.4898 | 307.5445 | 307.5555 | 307.2806 | **306.0984** | 307.1866 |
| Abbreviations: H-CHOP: Hanlikang, Cyclophosphamide, Doxorubicin, Vincristine, and Prednisone; R-CHOP: MabThera, Cyclophosphamide, Doxorubicin, Vincristine, and Prednisone; OS, overall survival; PFS, progression-free survival; AIC, Akaike information criterion; BIC, Bayesian information criterion. | | | | | | |
